# A pilot study on simultaneous stimulation of the primary motor cortex and supplementary motor area using gait-synchronized rhythmic brain stimulation to improve gait variability in post-stroke hemiparetic patients

**DOI:** 10.3389/fnhum.2025.1618758

**Published:** 2025-09-17

**Authors:** Kazuma Yamashita, Ruido Ida, Satoko Koganemaru, Mitsuya Horiba, Ippei Nojima, Tatsuya Mima, Yumie Ono, Sumiya Shibata, Takuya Hosoe, Hiromasa Tachiwa, Hiroaki Yamashita, Akihiro Itoh, Yuuki Murata, Masataka Fujita, Kaoru Kamimoto, Yoshino Ueki

**Affiliations:** ^1^Faculty of Health Sciences, Nihon Fukushi University, Nagoya, Japan; ^2^Department of Rehabilitation Medicine, Graduate School of Medical Sciences, Nagoya City University, Nagoya, Japan; ^3^Department of Rehabilitation Medicine, Osaka Medical and Pharmaceutical University, Osaka, Japan; ^4^The Graduate School of Core Ethics and Frontier Sciences, Ritsumeikan University, Kyoto, Japan; ^5^Department of Electronics and Bioinformatics, Meiji University, Tokyo, Japan; ^6^Department of Physical Therapy Institute for Human Movement and Medical Sciences (HIMMS), Niigata University of Health and Welfare, Niigata, Japan; ^7^Nagoya City University Mirai Kousei Hospital, Nagoya, Japan; ^8^Murata Hospital, Hoshokai Medical Corporation, Osaka, Japan; ^9^Department of Rehabilitation, Saishukan Hospital, Kitanagoya, Japan

**Keywords:** stroke, supplementary motor area, primary motor cortex, gait variability, gait-synchronized rhythmic brain stimulation

## Abstract

**Introduction:**

Gait impairment is a common and disabling consequence of stroke. While walking speed is a key indicator of recovery, gait variability is closely associated with fall risk and long-term functional decline. Previous studies have suggested that functional interaction between the supplementary motor area (SMA) and primary motor cortex (M1) plays a key role in post-stroke gait control. Rather than stimulating these regions independently, simultaneous activation of the SMA—critical for rhythm modulation and motor planning—and gait-synchronized stimulation of the M1—essential for motor execution—may offer enhanced benefits for gait stability.

**Objective:**

To assess the feasibility, safety, and preliminary effects of a combined brain stimulation intervention targeting the SMA and M1 on gait variability and balance in individuals with post-stroke hemiparesis.

**Methods:**

Sixteen individuals with stroke within 180 days after the onset, aged 40–90 years, who were able to walk on a treadmill were recruited in this study of multi-center, randomized, controlled pilot trial with a parallel-group design. Participants were randomly allocated to either an intervention group (*n* = 8) receiving 20 min of simultaneous transcranial direct current stimulation (tDCS) to the SMA and gait-synchronized rhythmic stimulation to the M1 during treadmill walking, or to a control group (*n* = 8) receiving sham stimulation. Both groups underwent 15 sessions of walking practice over 3 weeks. Primary outcomes were feasibility indicators including recruitment, retention, adherence and adverse events and preliminary estimates of effect on gait variability such as coefficient of variation for stride, stance, and swing times on the paretic side. Balance was assessed using the Mini-Balance Evaluation Systems Test (Mini-BESTest).

**Results:**

All 16 participants completed the intervention without adverse events, indicating high feasibility. The intervention group showed significantly reduced stride time variability on the paretic side and improved Mini-BESTest scores compared to the control group. A significant correlation was observed between reductions in gait variability and improvements in balance.

**Conclusions:**

This pilot trial supports the feasibility and safety of a combined SMA and M1 stimulation approach. Preliminary findings suggest potential benefits in reducing gait variability and improving balance after stroke, warranting further investigation in a definitive trial.

## 1 Introduction

In recent years, advancements in acute stroke care have led to a reduction in fatal brain injuries; however, this has resulted in a growing population of patients living with post-stroke sequelae. Accordingly, stroke management must now address not only survival outcomes but also functional recovery from the early stages of treatment. Upper and lower limb paralysis is observed in over 60% of stroke cases as an initial symptom, and the resulting decline in gait ability is a major factor contributing to difficulties in returning to independent home living (Yew and Cheng, 2015; Tai et al., 2022; Burton et al., 2018; Ruksakulpiwat et al., 2023). Gait ability refers to comprehensive indicator of walking function, including walking speed, endurance, and balance. Therefore, gait restoration is considered a paramount goal in stroke rehabilitation (Schmid et al., 2007; de Rooij et al., 2021).

Among the various indicators used to assess gait function in individuals with post-stroke hemiparesis, walking speed is one of the most critical. Numerous studies have reported that improvements in walking speed are closely associated with better performance in activities of daily living (ADL) (Schmid et al., 2007; Robinson et al., 2011; Gastaldi et al., 2015; Park and Kim, 2016). However, although conventional rehabilitation improves walking speed, many stroke survivors continue to experience a significantly higher risk of falls than healthy individuals, particularly within the first 6 months after discharge (Wong et al., 2016). Although walking speed is frequently used as a proxy for mobility, it may not be sufficient to evaluate the performance of balance and the impact of falls after discharge from in-patient stroke rehabilitation. While fall risk is often evaluated based on balance impairments or previous falls, these indicators may not capture subtle motor instabilities. Gait variability, in contrast, provides a more sensitive measure of such motor control deficits that may underlie fall risk. Gait variability can be categorized into temporal variability such as stride time, stance time and spatial variability such as step width, step length. Emerging evidence suggests that specific spatiotemporal gait variability parameters—particularly stride time variability and stance time variability—are strongly correlated with fall risk and can be a predictor of falls risk (Maki, 1997; Schinkel-Ivy et al., 2016; Toebes et al., 2012).

Motor impairments following stroke are often attributed to damage in the M1,SMA, or their associated descending pathways, such as the corticospinal tract (CST) and reticulospinal tract (RST). Damage to these cortical regions and neural circuits can profoundly disrupt a broad spectrum of motor functions, including rhythm regulation, postural control, and voluntary movement execution (Takakusaki, 2017). In addition to their well-established roles in upper limb motor control, the M1 and SMA also contribute to gait control by coordinating descending motor commands to the lower limbs and modulating interlimb coordination. Rhythm regulation in gait is also thought to be mediated by brainstem-spinal networks, particularly the central pattern generator (CPG), which generates basic rhythmic locomotor patterns. The SMA may influence these networks by providing preparatory signals and higher-order timing cues.

The supplementary motor area (SMA) contributes to anticipatory postural adjustments (APAs), which are essential for initiating and stabilizing gait transitions such as step initiation (Jacobs et al., 2009). APAs prepare the body by generating counterbalancing forces in anticipation of voluntary movement, reducing sway and enabling smoother gait initiation. In addition, the SMA plays a role in rhythm modulation, defined as the adjustment of temporal aspects of gait including step timing and cadence. This fine-tuning of time variability in gait is thought to involve both feedforward mechanisms of motor planning, prediction and feedback mechanisms of sensory inputs during locomotion. In contrast, M1 play a role in executing voluntary motor commands, resulting in controlling overall gait stability, defined as unintended medial-lateral or anterior-posterior displacement of the center of mass during ambulation (Kurz et al., 2012). Therefore, the combined approach both SMA and M1 could be critical for improving gait variability and stability in individuals with post-stroke hemiparesis (Kurz et al., 2012).

Recent developments in non-invasive brain stimulation (NIBS) techniques, such as tDCS and transcranial alternating current stimulation (tACS), have introduced new possibilities for promoting motor function recovery by modulating cortical excitability and facilitating neuroplasticity. These approaches have shown promise in improving motor performance after stroke (Meng et al., 2024). In our previous study, we developed a novel method called gait-synchronized rhythmic brain stimulation, in which tACS over M1 is synchronized with the patient’s gait cycle by using signal from a heel pressure sensor attached to the paretic foot (Kitatani et al., 2020; Koganemaru et al., 2019). The onset of tACS is triggered by a heel-strike event detected by a pressure sensor on the paretic foot, and then the peak of waveform is applied to subject, coincides with the onset of the swing phase in their gait. This method allows for phase-specific modulation of motor cortical activity in individual gait, and has been previously validated in our earlier studies (Kitatani et al., 2020; Koganemaru et al., 2019). Peripheral nerve electrical stimulation (PES) was also combined to assist dorsiflexion of the paretic ankle during gait. PES facilitates motor output by enhancing sensory afferent input, which can increase cortical excitability and promote activity-dependent plasticity. When applied to the paretic ankle dorsiflexors during gait, PES can support adequate foot clearance and improve gait symmetry and efficiency. This combined intervention resulted in significantly greater improvements in walking speed compared to PES-based gait training alone (Kitatani et al., 2020; Koganemaru et al., 2019). Furthermore, it has been reported that tDCS applied to the SMA can enhance movement-related preparatory potentials and improve postural adjustments (Nomura and Kirimoto, 2018). Based on previous studies, the combined approach both SMA and M1 could be critical for improving gait variability and stability in individuals, however, prior research has largely focused on the stimulation of these areas in isolation. To date, the simultaneous approach of gait-synchronized rhythmic stimulation of M1 while concurrently activating SMA by tDCS has not been thoroughly investigated. Given the known the different contribution to gait variability and stability between these regions, this combined approach may induce potential to improve gait variability and stability, beyond the effects of individual stimulation sites.

Despite promising findings in prior studies, the feasibility, safety, and potential synergistic effects of this simultaneous stimulation approach remain uncertain. Before proceeding to a definitive randomized controlled trial (RCT), it is essential to explore whether this combined intervention is tolerable, implementable, and associated with improvements in gait variability.

This pilot trial aimed to evaluate the feasibility, safety, and preliminary efficacy of a combined intervention involving gait-synchronized rhythmic brain stimulation to the M1 and tDCS to the SMA in individuals with post-stroke hemiparesis. The trial was designed to address uncertainties regarding recruitment, adherence, and tolerability, and to provide preliminary estimates of its impact on gait variability and balance to inform the design of a future RCT.

## 2 Materials and methods

### 2.1 Participants

Sixteen patients diagnosed with either cerebral infarction including atherothrombotic, lacunar, and cardiogenic embolic types or cerebral hemorrhage were recruited from hospitalized patients in Saijukan Hospital (Saijukan Medical Corporation), Murata Hospital (Hosho-kai Medical Corporation), and Nagoya City University Mirai Kousei Hospital. Participants were recruited from June 2023 to February 2025. For recruitment of subjects, neither advertisements nor posters were used. The inclusion criteria were (1) age between 40 and 90 years and (2) within 180 days after the onset of stroke and (3) the ability to walk on a treadmill. Exclusion criteria were as follows; (1) history or comorbidity of other psychiatric or neurological disorders, (2) presence of metal implants such as intracranial clips, (3) implanted cardiac pacemaker, (4) history of epilepsy, (5) prior surgery involving the joints or spine, (6) comorbid cardiovascular or respiratory diseases posing exercise risk, (7) presence of unverified non-magnetic implants (e.g., neural stimulators, aneurysm clips, insulin pumps, stents, intraocular metals), (8) severe hearing impairment preventing communication, (9) pregnancy or suspected pregnancy, and (10) inability to comprehend the rehabilitation protocol. Physicians belonging to each hospital explained the informed consent to subjects who met the inclusion and exclusion criteria, and written informed consent was obtained from all subjects.

### 2.2 Experimental procedure

Basic information of the participants was summarized using mean ± standard deviation for normally distributed variables and median (interquartile range) for non-normally distributed variables, as determined by the Shapiro–Wilk test. This study protocol was approved by the Medical Ethics Committee of the Graduate School of Medicine, Nagoya City University (Approval No. 2023A001-2). This trial was registered in the Japan Registry of Clinical Trials (jRCT) under the number of jRCTs042230011 and the protocol can be accessed from the jRCT homepage. No changes to methods after pilot trial commencement were conducted. The trial was reported was contacted in accordance with the CONSORT 2010 extension for randomized pilot and feasibility trials and the TIDieR checklist for intervention description (Supplementary Data Sheet 1).

**FIGURE 1:**
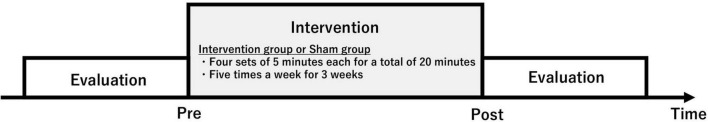
Experimental procedure. Participants were randomly assigned into two groups, real stimulation group or control group. Intervention group received gait-synchronized rhythmic brain stimulation to the M1 on the lesion side, and simultaneous tDCS to the SMA, whereas, the control group received sham stimulation both to M1 and SMA. The evaluation was conducted immediately before and after the intervention.

**FIGURE 2:**
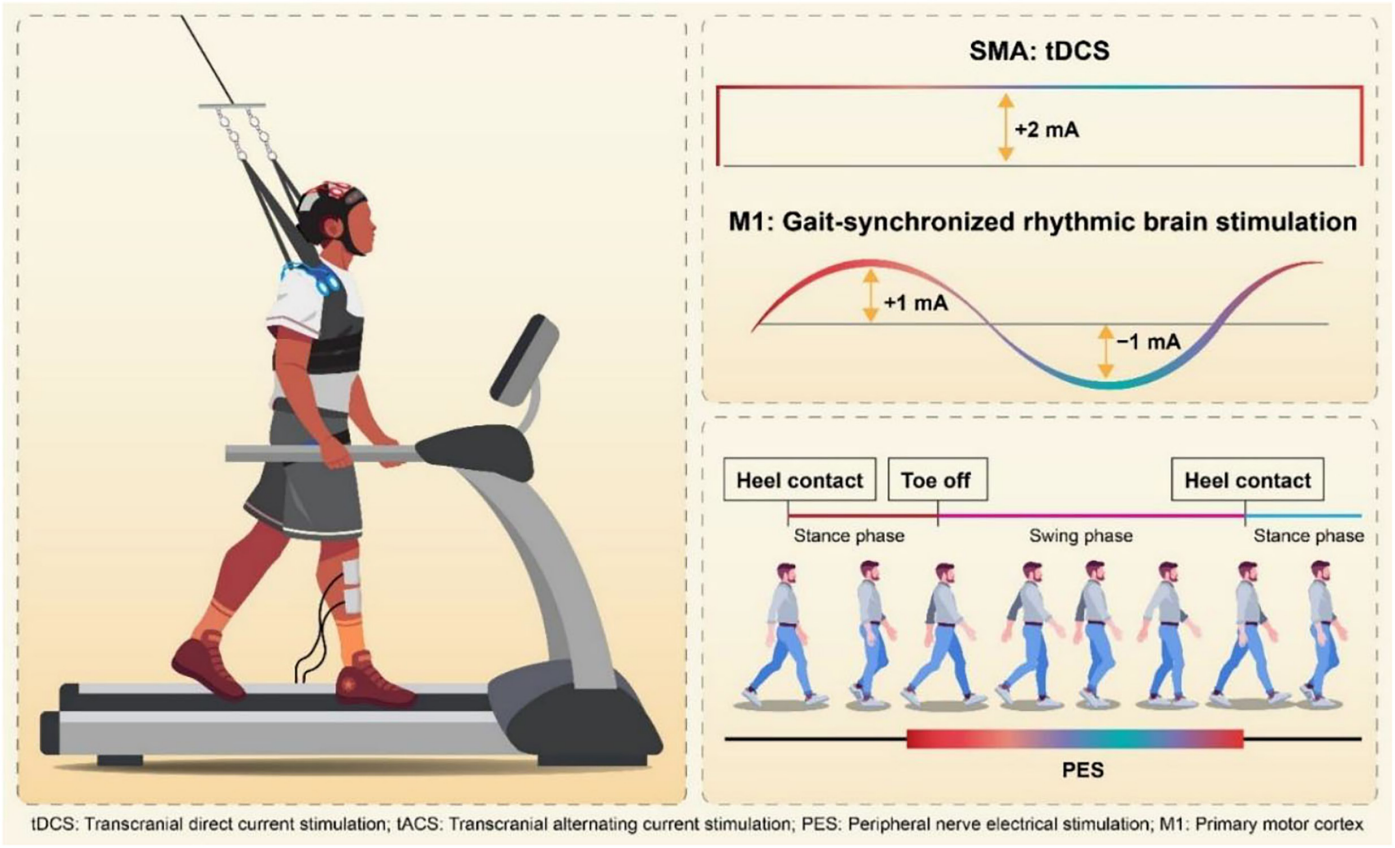
Simultaneous stimulation of the SMA and gait-synchronized M1. Gait-Synchronized Rhythmic Brain Stimulation, as in previous studies, used the optimal gait rhythm frequency (optimal frequency) for stimulation tailored to each patient. The stimulation waveform for tACS was delivered using a computer connected to a DC stimulator (in remote control mode), with a sinusoidal wave at an amplitude of 2 mA (ranging from –1 mA to + 1 mA). Each cycle of the current (rising from –1 mA to + 1 mA and falling from + 1 mA to –1 mA) was initiated at the time of heel contact on the paretic side, using a heel pressure sensor to ensure that the peak current intensity coincided with the onset of the swing phase. SMA stimulation was carried out using 2 mA tDCS (duration: 270 s, fade-in: 15 s, fade-out: 15 s). Simultaneously, peripheral functional electrical stimulation (FES) was applied to the tibialis anterior muscle, triggered by the heel pressure sensor to match the timing of the swing phase on the paretic side.

Participants were randomly assigned into two groups, real stimulation group or control group, based on a computer-generated randomization table created using Excel. The person who generated the random allocation sequence, confirmed the group of each subject and set the following stimulation pattern behind the examiner. Intervention group received gait-synchronized rhythmic brain stimulation to the M1 on the lesion side, and simultaneous tDCS to the SMA, whereas, the control group received sham stimulation both to M1 and SMA. Both groups received peripheral nerve electrical stimulation (PES) to assist ankle dorsiflexion on the paretic side. Treadmill walking training (PP-Tread, Molitoh Co., Ltd., Japan) comprised four sets of 5-minute walking with 3-minute rest intervals per session. Each participant completed one session per day, 5 days a week, for 3 weeks (15 total sessions). The evaluation was conducted immediately before and after the intervention (Figure 1). Specifically, baseline assessments were conducted on the same day or 1 day prior to the start of the first intervention session. If a participant demonstrated insufficient foot clearance during the swing phase, a harness system was used to partially unload body weight and assist leg swing as needed (Figure 2). At the time of randomization, the need for weight support (BWS) during intervention was not defined and after randomization, physician and physical therapist decided the use of BWS depend on insufficient leg swing in initial walking steps and high fall risk caused by imbalance. Before each session, the optimal frequency for the gait-synchronized brain stimulation was calculated based on prior studies (Kitatani et al., 2020; Koganemaru et al., 2019; Nojima et al., 2023). This frequency was derived by measuring the participant’s walking cadence during natural gait on a treadmill using a heel pressure sensor, and was adjusted throughout the intervention period. As natural gait, the subject walked in spontaneous and comfortable gait pattern without external cues or interventions. All participants received standard physical therapy and occupational therapy, and speech-language therapy was provided as needed. The total daily therapy time ranged from 120 to 180 min (40–60 min per session, three to four sessions per day).

### 2.3 Interventions

#### 2.3.1 Simultaneous stimulation of the SMA and gait-synchronized M1

**FIGURE 3:**
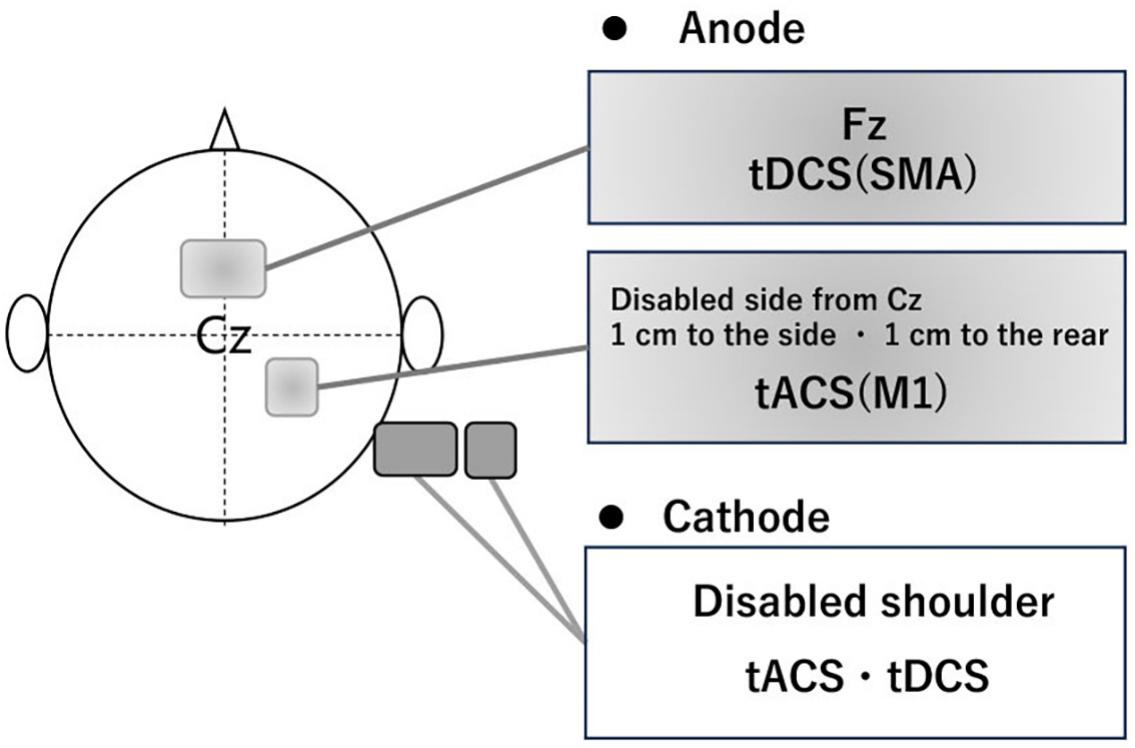
The position of electrode. To stimulate the M1 of the affected side, the electrode (3 × 3 cm) was placed using the international 10–20 system, with the anode positioned 1 cm lateral and 1 cm posterior from Cz and the cathode placed around the same side shoulder. To stimulate the SMA, the electrode (5 × 7 cm) had the anode positioned at Fz and the cathode placed around the same side shoulder.

Participants in the intervention group received gait-synchronized tACS to the M1 combined with tDCS to the SMA during treadmill gait training. Regarding M1 stimulation, tACS was delivered at each participant’s optimal frequency, which was continuously applied for 20 min based on the measurement prior to the intervention in each trial. The tACS waveform consisted of a 2 mA sinusoidal current (ranging from −1 mA to + 1 mA), generated by a computer-controlled DC stimulator (using DC-STIMULATOR PLUS, neuroConn GmbH, Germany). Stimulation was synchronized with the gait cycle: each stimulation cycle was triggered by a heel-strike event detected by a pressure sensor, with the peak of the waveform timed to coincide with the onset of the swing phase (Figure 2). Regarding SMA stimulation, anodal tDCS was applied at an intensity of 2 mA for 270 s, including a 15-second fade-in and a 15-second fade-out period. The electrodes placements were based on the 10–20 EEG system. For the M1 stimulation, a 3 × 3 cm anodal electrode was positioned at anterolateral and posterior to Cz (1 cm lateral and 1 cm posterior to Cz) and for the SMA stimulation, a 5 × 7 cm anodal electrode at Fz (Weiss et al., 2013; Lotze et al., 2003), with the cathode placed on the ipsilateral shoulder, independently (Figure 3).

This simultaneous stimulation of the SMA and gait-synchronized M1 was applied during overall treadmill walking training. Safety monitoring included asking participants before, during, and after each session about any unusual sensations (e.g., phosphenes or scalp itching). The electrode contact areas were also checked for any skin reactions.

#### 2.3.2 Sham stimulation

As a sham stimulation in the control group, electrodes were placed in the same positions as in the real stimulation without real current delivered throughout all sessions.

#### 2.3.3 Peripheral nerve stimulation

As a peripheral nerve stimulation, electrodes were placed on the tibialis anterior muscle, distal-anterior to the fibular head (device: SEN-3401, Nihon Kohden Corp., Japan). PES was set at approximately 10 mA, with a pulse width of 250–500 μsec and a stimulation frequency of 40 Hz. The stimulation was triggered by signals from a pressure sensor attached to the heel of the paretic foot. The “on” signal corresponded to heel contact (stance phase onset), and the “off” signal corresponded to heel lift (stance phase offset). PES was delivered during the period from heel lift to the next heel contact, covering most of the swing phase. PES was applied to assist ankle dorsiflexion on the paretic side in both groups.

### 2.4 Evaluation and outcomes

#### 2.4.1 Feasibility outcomes

The primary feasibility outcomes included:

(1)    Recruitment rate – the number of eligible participants successfully enrolled in each hospital between June 2023 and February 2025;
(2)    Adherence to the intervention protocol – defined as the proportion of participants who completed all 15 sessions of the intervention over 3 weeks;
(3)    Retention rate – the number of participants who completed the post-intervention assessment;
(4)    Safety – evaluated by recording any adverse events (e.g., dizziness, headache, discomfort, skin reactions) during or after stimulation sessions. All phenomenon were reported after the intervention began or after it ended in each session, until 1 month after the end of intervention.

#### 2.4.2 Preliminary efficacy outcomes

Gait, physical function, subjective measures, and activities of daily living (ADL) were evaluated immediately before and after 3-week intervention.

Gait function was assessed using 10-meter walk test, 6-minute walk test, gait variability including the coefficient of variation (CV) of stride time, stance time, and swing time on the paretic side, calculated from 20 steps during natural gait using heel pressure sensors sampled at 100 Hz. CV was calculated using the following formula: CV = (Standard Deviation/Mean) × 100 (Patel et al., 2022; Lim et al., 2023). Physical function was evaluated using Fugl-Meyer Assessment (FMA) for upper and lower extremities (Fugl-Meyer et al., 1975), Sit-to-Stand test and Mini-Balance Evaluation Systems Test (Mini-BESTest). Subjective measures included Modified Falls Efficacy Scale (mFES) and Numerical Rating Scale (NRS) for walking difficulty, ranging from 0 (“very difficult to walk”) to 10 (“very easy to walk”) (Fugl-Meyer et al., 1975). ADL were assessed using Functional Independence Measure (FIM). All outcome measures are summarized in Table 1.

**TABLE 1:** Evaluation items.

The details of evaluation items
**Gait function**
Gait speed in10 m walk test
6-minute walk test
Gait variability
(coefficient of variation (CV) for swing, stance phase time and stride time variability on the paretic side)
**Physical function**
Fugl-Meyer Assessment (Fugl-Meyer et al., 1975)
Sit to stand
Mini-BESTest
**Subjective evaluation**
Modified Falls Efficacy Scale (mFES)
Numerical Rating Scale (NRS)
**Activity of daily living**
Functional Independence Measure (FIM)

As a gait parameter, we measured 10-meter walking speed, gait variability and 6-minute walk test. Gait variability was assessed using the coefficient of variation (CV) for swing and stance time on the paretic side and stride time variability on the paretic side. Physical function was evaluated using the Fugl-Meyer Assessment, the Sit-to-Stand test, and the Mini-BESTest. Subjective measures included the Modified Falls Efficacy Scale (mFES) and the Numerical Rating Scale (NRS). ADL performance was assessed using the Functional Independence Measure (FIM).

#### 2.4.3 The walking speed and stimulation frequency during intervention

In order to examine the mean changes of walking speed and stimulation frequency by intervention, the averaged treadmill speed and corresponding frequency of tACS for each session (sessions 1, 3, 6, 9, 12, and 15) were recorded throughout the intervention period. Before starting intervention in each session, the preferred walking speed was determined based on natural treadmill walking at a comfortable pace without external cues and was adjusted until they felt comfortable and natural walk. The frequency of tACS was determined for each participant prior to each session by measuring the cadence based on the preferred walking speed described before, and the stimulation frequency was set to synchronize with the individual’s gait cycle using heel pressure sensors on paretic side.

### 2.5 Statistical analysis

Comparisons between the intervention and control groups were made for gait, physical function, subjective measures, and ADL. The Shapiro-Wilk test was used to assess the normality of the data. The Shapiro–Wilk test showed that the coefficient of variation (CV) of stride time and stance time, 6-minute walk test, and Mini-BESTest followed a normal distribution.

Regarding gait function, each parameter was analyzed using a linear mixed model ANOVAs to examine the effects of condition (real vs sham) and time (pre vs post), while adjusting for potential confounding variables if needed. Subject was specified as a random intercept to account for within-subject correlations due to repeated measures. Bonferroni procedures were used to correct for multiple comparisons in the *post hoc* analysis of gait function. The Satterthwaite approximation was applied to estimate degrees of freedom for fixed effects tests. For non-normally distributed data, we calculated the log-transformed rate of change for each participant. The distributions of the log-transformed change rates were compared using Mann–Whitney U tests. Within-group comparisons were performed using the Wilcoxon signed-rank test, and correlation analyses of the logarithmic rate of change in gait variability with the Sit-to-Stand test, as well as with the Mini-BESTest and FMA upper limb scores, were conducted using Pearson’s or Spearman’s correlation coefficients depending on the data type. In addition, changes in average treadmill speed between sessions 1 and 15 were evaluated using a two-way ANOVA.

Effect sizes were calculated for both parametric and non-parametric analyses. For ANOVA results, partial eta squared (η^2^p) was computed as SS_effect/(SS_effect + SS_error), where SS represents the sum of squares. Following conventional benchmarks reported by Cohen (1988), η^2^p values of 0.01, 0.06, and 0.14 were interpreted as small, medium, and large effects, respectively. For within-group pre–post comparisons, Cohen’s d for paired samples was calculated as the mean of the difference scores divided by the standard deviation of the difference scores, with thresholds of 0.20, 0.50, and 0.80 indicating small, medium, and large effects, respectively. For non-parametric tests (Mann–Whitney U), the effect size r was calculated as Z/√N, where Z is the standardized test statistic and N is the total number of observations. Based on Cohen’s guidelines, r values of 0.10, 0.30, and 0.50 were interpreted as small, moderate, and large effects, respectively.

All statistical analyses were performed using IBM SPSS Statistics Version 29, and a significance level of α = 0.05 was set for all tests. The sample size was not determined based on statistical power to detect treatment effects. It was selected to provide sufficient information on feasibility parameters to inform the design of a subsequent definitive trial. However, in this pilot study, gait variability was treated as exploratory primary outcomes to assess preliminary efficacy. *Post hoc* power analysis was not conducted due to the small sample size (*n* = 8 per group) and the lack of reliable prior estimates of effect sizes for these outcomes, which would limit the interpretability and validity of such analysis.

## 3 Results

### 3.1 Patient characteristics

All screened potential participants were finally enrolled in this study. Participants were randomly assigned to two groups: the intervention group (*n* = 8, age: 56.25 ± 10.87 years, sex: 7 males/1 female) and the control group (*n* = 8, age: 62.87 ± 15.76 years, sex: 4 males/4 females) (Supplementary Figure 1). All participants completed the study protocol without any adverse events, such as dizziness or discomfort, during the intervention. Although safety monitoring included asking participants before, during, and after each session about any unusual sensations, no one noticed which group they participate in until finishing all sessions. In the control group, two participants required BWS during treadmill walking due to insufficient leg swing in initial walking steps. The unloading amount was 10–15 kg, corresponding to approximately 15–20% of their body weight. No significant differences were observed between groups in age, body mass index (BMI), or duration from stroke onset to intervention. The height and body weight were significantly different between two groups (Table 2).

**TABLE 2:** Patients characteristics.

Data	Unit	Real stimulation (*n* = 8)	Control (*n* = 8)	*p*-value
Sex	Men/women	7/1	4/4	
Age	Year	56.25 ± 10.87	62.87 ± 15.76	0.344
Height	cm	165.92 ± 7.85*	156.56 ± 8.53*	0.039
Body weight	kg	65.61 ± 12.58*	50.13 ± 9.41*	0.015
Body mass index	kg/m2	23.70 ± 3.20	20.54 ± 4.24	0.115
Time from stroke onset	Days	58.5 (42.25–96.25)	48.0 (43.5–67.75)	0.574
Disability type	Infarction/hemorrhage	5/3	6/2	
Hemiparetic side	Right/left	3/5	5/3	
Gait ability	Cane/free hand	4/4	6/2	
Orthosis	Yes/no	3/5	3/5	

Basic information of the participants was presented with mean ± standard deviation for items that followed a normal distribution and median (interquartile range) for items that did not follow a normal distribution, as determined by the Shapiro-Wilk test. For group comparisons, an independent *t*-test and Mann-Whitney U test were performed. As a result, significant differences were found between the intervention group and the control group in terms of height and weight, but no significant differences were observed for other items (*p < 0.05).

### 3.2 Feasibility outcomes

Sixteen eligible participants were recruited eleven from Saijukan Hospital, two from Murata Hospital, and three from Nagoya City University Mirai Kousei Hospital between June 2023 and February 2025. All participants completed all fifteen sessions without dropout, achieving 100% adherence. They completed the full 20 min of walking training in every session without interruption or early termination and the post intervention assessment. No adverse events were reported during the intervention period or the one-month follow-up. These results indicate high feasibility and acceptability of the simultaneous SMA and M1 stimulation protocol in the present study.

Regarding the protocol, no changes were made to the prespecified outcome assessments or measurement procedures after trial commencement. No formal progression criteria were established to determine whether or how to proceed to a future definitive RCT.

### 3.3 Efficacy of intervention

**FIGURE 4:**
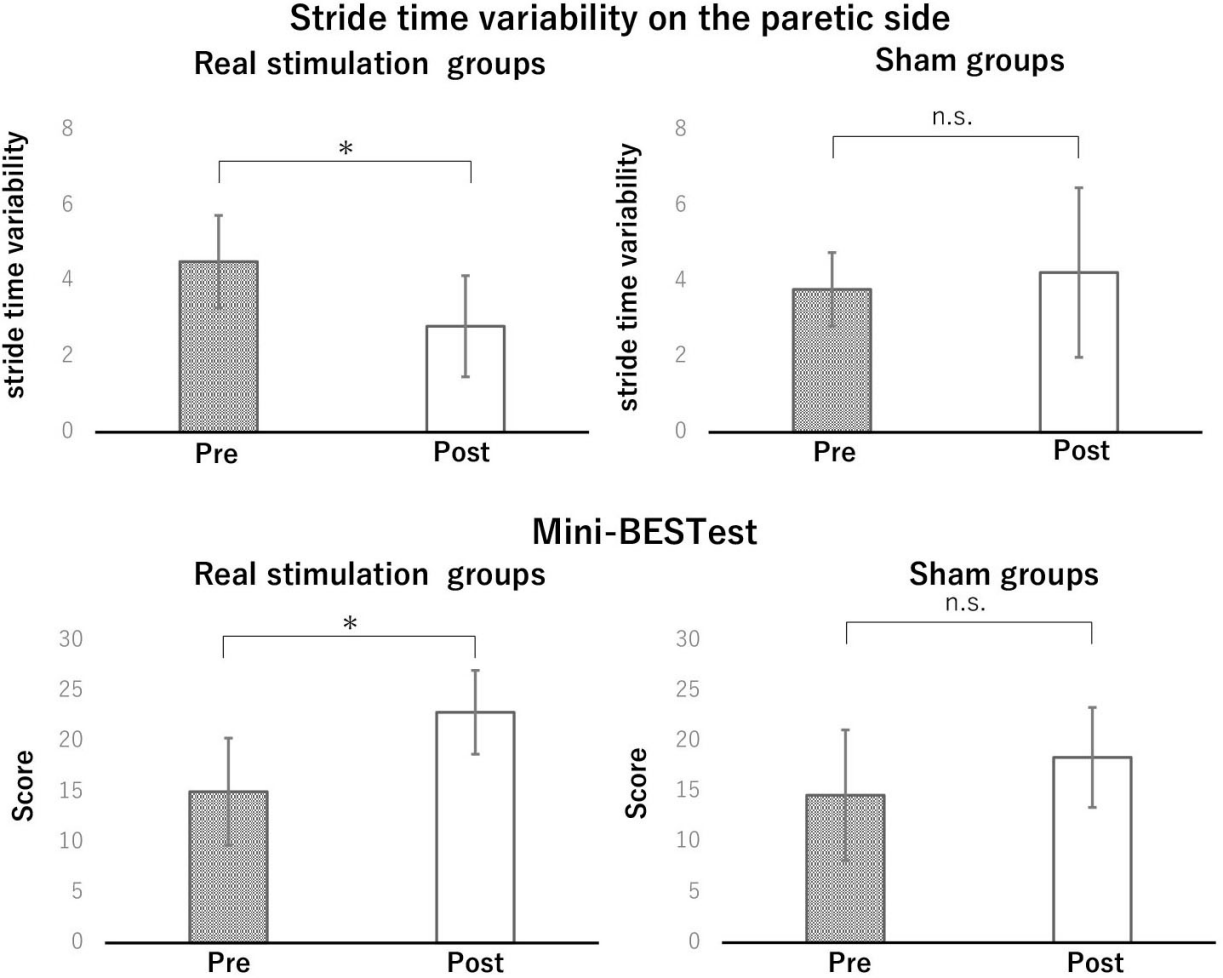
The effect of intervention in the stride time variability on the paretic side and mini-BESTest. In the stride time variability on the paretic side and mini-BESTest, *post hoc* analysis revealed that the CV of stride time on the paretic side and mini-BESTest were significantly improved after the real stimulation. No significant differences were found for the other items (*p < 0.05).

Because of significantly difference in height and body weight, each parameter of gait function was analyzed using a linear mixed-effects model to examine the effects of condition and time, while adjusting for potential confounding variables (height and weight). The model included condition, time, and their interaction as fixed effects, with height and weight included as covariates. For CV of stride time on the paretic side, the linear mixed model measure ANOVAs showed significant condition × time interaction [*F*(1,14) = 6.27, *p* = 0.025*, η^2^p = 0.309, large], but no significant main effects of condition (*F*(1,12) = 0.21, *p* = 0.66, η^2^p = 0.017, small) or time [*F*(1,14) = 2.15, *p* = 0.16, η^2^p = 0.133, medium]. Height and weight did not significantly contribute to the model (*p* = 0.66 and *p* = 0.78, respectively). *Post hoc* analysis revealed that the CV of stride time on the paretic side was significantly decreased after the real stimulation (*p* = 0.014*, Cohen’s d = -1.10, large), while no significant change was observed after the sham (*p* = 0.48, d = 0.24, small) (Figure 4). For CV of swing time on the paretic side, the linear mixed model measure ANOVAs revealed a significant main effect of time [*F*(1,14) = 66.21, *p* < 0.0001, η^2^p = 0.825, large], whereas, no significant main effect of condition (*p* = 0.801, η^2^p = 0.002, negligible) or condition × time interaction (*p* = 0.779, η^2^p = 0.003, negligible) was observed. For CV of stance time on the paretic side, the analysis revealed no significant main effect of condition (*F*(1,12) = 0.0006, *p* = 0.981, η^2^p < 0.001, negligible), time [*F*(1,14) = 1.97, *p* = 0.182, η^2^p = 0.123, medium], or condition × time interaction [*F*(1,14) = 2.51, *p* = 0.135, η^2^p = 0.152, large]. For the Mini-BESTest, the linear mixed model measure ANOVAs revealed a significant condition × time interaction [*F*(1,14) = 6.27, *p* = 0.025, η^2^p = 0.309, large], without significant main effects of condition [*F*(1,12) = 0.21, *p* = 0.66, η^2^p = 0.017, small] or time [*F*(1,14) = 2.15, *p* = 0.16, η^2^p = 0.133, medium]. *Post hoc* analysis revealed a significant improvement in the real stimulation (*p* = 0.012*, *d* = 1.05, large), while no significant change was observed in the sham group (*p* = 0.60, *d* = 0.14, small). Regarding gait speed in 10-meter walking and 6-minute walk test, the analysis revealed a significant main effect of time [*F*(1,14) = 25.4, *p* = 0.0002, η^2^p = 0.645, large and *F*(1,14) = 18.50, *p* = 0.0007, η^2^p = 0.569, large, respectively], indicating that gait speed significantly increased from pre- to post-intervention in both groups. No significant main effect of condition (*p* = 0.54, η^2^p = 0.030, small and *p* = 0.46, η^2^p = 0.035, small, respectively) or condition × time interaction (*p* = 0.17, η^2^p = 0.103, medium and 0.23, η^2^p = 0.084, medium, respectively) was observed (Table 3).

**TABLE 3:** The effect of intervention to the gait and physical functions.

Data	Real stimulation (*n* = 8)		Control (*n* = 8)	
	Pre	Post	Pre	Post
Gait speed (m/s)	0.89 ± 0.29	1.20 ± 0.37	0.74 ± 0.34	0.91 ± 0.35
Swing phase time variability	5.10 ± 1.53	5.70 ± 3.28	6.11 ± 2.33	6.14 ± 1.48
Stance phase time variability	11.64 ± 7.80	6.33 ± 1.43	8.42 ± 3.08	8.79 ± 5.15
Stride time variability	4.51 ± 1.18	2.80 ± 1.29*	3.77 ± 0.94	4.22 ± 2.16
6-minute walk test (meter)	258.37 ± 104.49	340.00 ± 124.12	215.37 ± 100.67	260.12 ± 107.37
Mini-BESTest	15.00 ± 5.31	22.87 ± 4.15*	14.62 ± 6.47	18.37 ± 4.95

The *P* values were produced using *post hoc* analysis pre- and post-intervention. As a result, significant differences were found in stride time variability and Mini-BESTest (*p < 0.05), but no significant differences for other items.

The Mann–Whitney U test was used for the NRS, FMA, sit-to-stand test, mFES, and FIM motor score due to non-normal distribution. A significant difference was found in the logarithmic rate of change of NRS, with the real stimulation group exhibiting a larger reduction than the control group (*U* = 55.5, *p* = 0.012, *r* = 0.62, large effect size). No significant differences were observed between groups for FMA (*U* = 49.0, *p* = 0.063, *r* = 0.45, moderate), sit-to-stand performance (*U* = 49.0, *p* = 0.063, *r* = 0.45, moderate), mFES (*U* = 39.5, *p* = 0.460, *r* = 0.20, small), or FIM motor score (*U* = 33.0, *p* = 0.958, *r* = 0.03, negligible).

**FIGURE 5:**
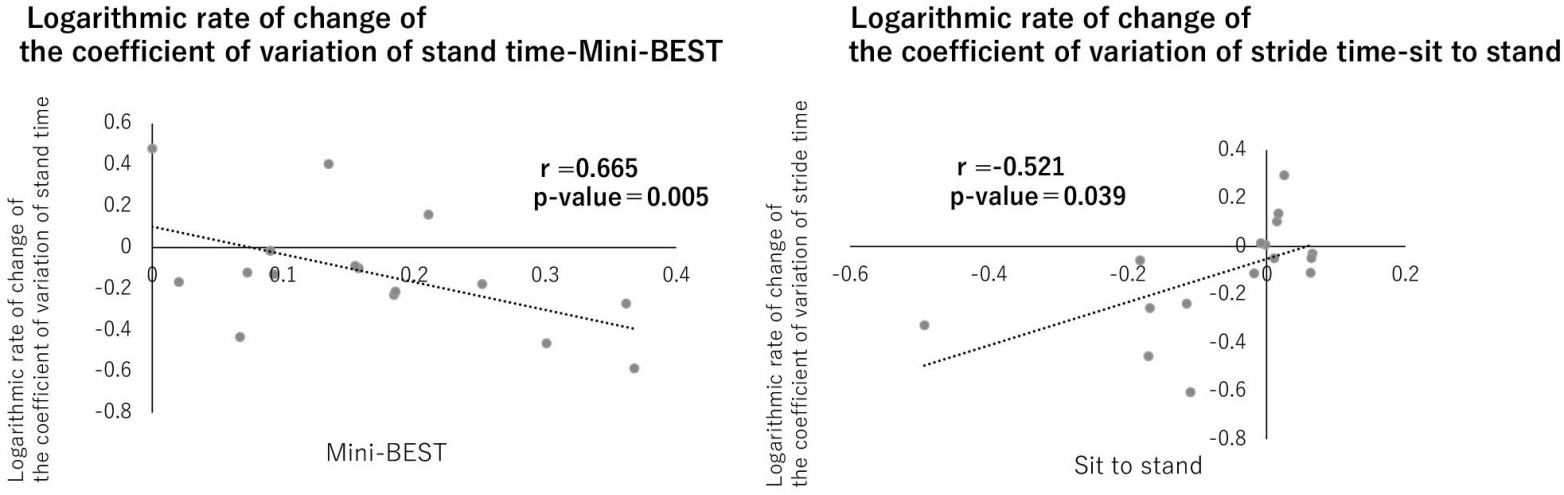
Correlation between gait variability and physical functions. A correlation was observed between the logarithmic change rates of the stance phase time variability coefficient and Mini-BEST and between the stride time variability coefficient and sit-to-stand. No significant differences were found for the other items (*p* < 0.05).

Correlation analysis revealed a significant positive relationship between CV of stance time on the paretic side and the Mini-BESTest, and a significant negative relationship between CV of stride time on the paretic side and the sit-to-stand test (*p* < 0.05, Figure 5), while no significant correlations were found between upper limb scores of FMA and any gait measurements. The intensity of PES increased from 9.75 ± 0.68 mA to 12.2 ± 0.66 mA over the 15 sessions in the intervention group, and from 9.85 ± 0.70 mA to 11.8 ± 0.85 mA in the control group. However, the small sample size in this pilot study (*n* = 8 in each group) may have limited the statistical power to detect differences.

### 3.4 The changes of walking speed and frequency of tACS in each session by intervention

**FIGURE 6:**
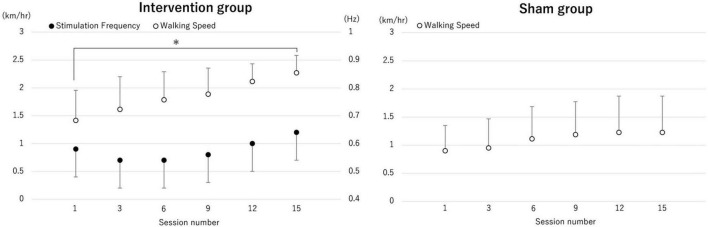
The change of averaged treadmill speed and the transcranial alternating current stimulation (tACS) frequency. The average values of treadmill speed in each session (session number 1, 3, 6, 9, 12 and 15) and tACS frequency for each session are shown. The treadmill walking speed in session 15 after the real brain stimulation was significantly increased compared with session 1, but not after sham stimulation. *p < 0.05.

Rhythmic brain stimulation synchronized with the gait cycle showed progressive changes over time, including alterations in stimulation frequency and walking speed. The average stimulation frequency for the intervention group increased from 0.58 ± 0.08 Hz in the first session to 0.60 ± 0.13 Hz in the 15th session. Regarding the change of treadmill walking speed, two-way ANOVA showed a significant main effect of time (p < 0.001) and interaction (p = 0.024). Post hoc analysis revealed that the treadmill walking speed in 15th after the real brain stimulation was significantly increased compared with 1st (p = 0.002), but not in the control group. The mean value of treadmill walking speed in real brain stimulation increased from 1.41 ± 0.50 km/h to 2.20 ± 0.30 km/h. In the control group, treadmill speed increased from 0.90 ± 0.45 km/h to 1.22 ± 0.64 km/h (Figure 6).

## 4 Discussion

### 4.1 Feasibility of the intervention

This pilot trial demonstrated high feasibility of the combined intervention involving gait-synchronized rhythmic brain stimulation to the M1 and tDCS to the SMA. All 16 participants successfully completed all 15 intervention sessions (100% adherence) without any adverse events such as dizziness, headaches, or skin irritation. No participants withdrew from the study, and all tolerated the stimulation protocols well. These findings suggest that the intervention protocol was well accepted and implementable in individuals with post-stroke hemiparesis. These results support the feasibility of conducting RCT using this combined stimulation approach.

### 4.2 Efficacy and mechanism of simultaneous stimulation of M1 and SMA

This pilot trial revealed the feasibility, safety, and preliminary efficacy of a combined intervention involving gait-synchronized rhythmic brain stimulation to the M1 and tDCS to the SMA in individuals with post-stroke hemiparesis. In the present study, the condition × time interaction for CV of stride time on the paretic side and Mini-BESTest demonstrated a large effect size, indicating that the active stimulation produced a substantial reduction in gait variability and stability. The within-group analysis further supported this finding, with a large improvement in the real stimulation group. Taken together, the simultaneous stimulation significantly induced meaningful changes in both gait stability and balance control in individuals with post-stroke hemiparesis. These large effect sizes, observed in the context of a pilot trial, provide important information for estimating sample sizes and setting clinically relevant outcome thresholds in future definitive randomized controlled trials. Furthermore, significant correlations were observed between the CV of stance time and the Mini-BESTest, as well as between the CV of stride time and the sit-to-stand test. These findings suggest that simultaneous stimulation of the SMA and M1 may contribute to improvements in both gait variability and balance function in post-stroke hemiparetic patients.

Importantly, gait stability was improved through rhythmical stimulation of M1 while concurrently activating the SMA via tDCS. This effect may be attributed to two potential physiological mechanisms: (1) an additive modulation of the central pattern generator (CPG) through dual-site stimulation, or (2) enhanced cortico-cortical coupling between the SMA and M1. CPG plays a fundamental role in generating rhythmic locomotor patterns independent of supraspinal input (Grillner, 2006). Our findings suggest that simultaneous stimulation of the SMA and M1 may modulate cortical inputs that interact with the CPG, potentially enhancing rhythm generation and gait stability in individuals with post-stroke hemiparesis. This is consistent with previous reports indicating that supraspinal structures, including the SMA, can influence CPG activity to fine-tune gait patterns (Nielsen, 2003). This novel approach—simultaneously stimulating multiple brain regions using non-invasive brain stimulation (NIBS)—was well-tolerated with no adverse events reported, indicating high safety.

The SMA is known to be involved in anticipatory postural adjustments, rhythm control, motor planning, movement automation, and bilateral coordination, all of which are crucial for gait and balance (Jacobs et al., 2009). Previous studies have highlighted its role in regulating step length and timing (Jacobs et al., 2009; Kurz et al., 2012). The M1, responsible for executing voluntary motor commands and adjusting muscle output, plays a central role in maintaining gait rhythm and stability (Kurz et al., 2012). Therefore, not only M1 stimulation alone but also the functional interaction between the SMA and M1 appears essential for effective gait rehabilitation following stroke (Kurz et al., 2012). In this study, the simultaneous stimulation of these areas may have facilitated rhythm adjustment during walking, leading to more stable stride time and improved gait variability. Additionally, the motor plans formulated in the SMA may have been more effectively transmitted and executed via the M1, resulting in smoother and more efficient gait patterns. Furthermore, since no significant correlations between upper limb scores of FMA and each gait measurement was found, upper limb dysfunction did not directly influence the change of gait variability observed in this study.

### 4.3 Evaluation of outcome measures

The outcome measures selected in this pilot trial were appropriate for gait function including gait variability, physical function of Mini-BESTest and subjective evaluation of NRS in individuals with post-stroke hemiparesis. However, both the FMA and FIM motor subscale showed relatively high baseline values in several participants, indicating a possible ceiling effect that may have limited their responsiveness to further improvement. Similarly, the mFES demonstrated clustering of scores near the upper range, reflecting reduced sensitivity to detect subtle changes in balance. Based on these, complementary metrics such as instrumented gait analysis, balance confidence scales, or individualized goal attainment scaling may provide more nuanced assessments of intervention effects, especially in patients with mild to moderate hemiparesis. Nevertheless, the present outcome set offered clinically meaningful insights into functional changes and was broadly suitable for this population in the context of a pilot feasibility trial.

### 4.4 Clinical implication

Correlations between gait variability indices and balance assessments (e.g., sit-to-stand and Mini-BES Test) further support the relevance of this intervention in addressing fall risk in stroke survivors. Previous studies have also reported associations between SMA activity and postural control (Mihara et al., 2012; Fujimoto et al., 2014), which aligns with the observed improvements in both static and dynamic balance measures. Gait variability, particularly stride time variability, has been shown to be a strong predictor of fall risk in older adults and individuals with post-stroke (Maki, 1997; Schinkel-Ivy et al., 2016; Toebes et al., 2012). In this study, the stride time variability and Mini-BESTest scores in the intervention group significantly improve by exceeded 1.5% as absolute value and averaged 8 points, respectively, which is comparable to be greater than the minimal clinically important difference reported in previous studies of post-stroke hemiparesis. Notably, gait variability and balance assessments—such as the sit-to-stand test and the Mini-BESTest—have been previously associated with fall risk in patients with stroke (Maki, 1997; Schinkel-Ivy et al., 2016; Toebes et al., 2012). These preliminary findings suggest potential benefits in reducing gait variability and improving balance after stroke, warranting further investigation including fall prevention in a definitive trial. Moreover, in this pilot trial, although some outcomes did not reach statistical significance due to the limited sample size, the reported effect sizes provide valuable insights into the potential clinical efficacy of the intervention. For example, the NRS demonstrated a large effect (*r* = 0.62), suggesting a meaningful benefit of simultaneous stimulation on symptom relief. Similarly, the FMA lower extremity score showed a medium-to-large effect size (*r* = 0.45), indicating a potential improvement in motor function that may become statistically significant in a larger, adequately powered trial. The present findings support the feasibility of the intervention and provide preliminary evidence of its potential therapeutic impact, which justifies proceeding to a future definitive RCT with an appropriately powered sample size. Based on this pilot study, the stride time variability is the most candidate of primary outcome measure for further study.

### 4.5 Limitations

Nevertheless, this study has several limitations. First, the absence of M1-only and SMA-only stimulation groups means that we cannot isolate the specific contribution of each stimulation site, which limits the causal interpretation of the combined stimulation effects observed in this study. Although previous studies have reported improvements in gait speed with M1-targeted rhythmic stimulation (Kitatani et al., 2020; Koganemaru et al., 2019), they did not evaluate gait variability, and the specific effects of SMA-only stimulation also remain unclear (Jacobs et al., 2009; Kurz et al., 2012). Future studies should include M1-only, SMA-only, and combined M1 + SMA stimulation groups, using standardized intervention protocols and accounting for stroke chronicity to clarify the individual and additive effects of each stimulation site. Second, the long-term sustainability of the effects was not evaluated, warranting follow-up studies. Third, the sample size in each group was small (*n* = 8), resulting in insufficient statistical power and an increased risk of Type II errors. Fourth, there was imbalance in gender distribution (11 males, 5 females). In addition, at the time of randomization, the need for BWS during intervention was not determined, causing imbalance of use for BWS. Future studies should be assessed the use of BSW more structural criteria before randomization and include the stratification factor for statistical analysis. Fifth, the age range of the participants was relatively broad (40–90 years). Age-related changes to not only physical and cognitive functions but neuroplasticity in cortical excitability and sensory integration may affect the efficacy of tDCS and tACS interventions. Although no subgroup analysis of gender and age differences were conducted due to the small sample size, these differences might affect the outcome especially related to gait functions. Future studies with larger sample sizes will be necessary to confirm these preliminary findings with appropriate statistical adjustments.

## 5 Conclusion

This pilot study suggests that simultaneous stimulation of the SMA and gait-synchronized M1 may reduce gait variability, potentially contributing to improved balance in post-stroke hemiparetic patients without any adverse effect. However, since this study include small sample size, the effect of clinical impact such as avoid the risk of fall remains unclear. Given the feasibility nature of this pilot study, the findings should be interpreted as preliminary and will serve as a basis for designing a fully powered RCT with sufficient statistical power and appropriate comparator arms to validate these preliminary observations.

## Data Availability

The original contributions presented in this study are included in this article/Supplementary material, further inquiries can be directed to the corresponding authors.
