# Supplementary figures and images for: A pilot study on simultaneous stimulation of the primary motor cortex and supplementary motor area using gait-synchronized rhythmic brain stimulation to improve gait variability in post-stroke hemiparetic patients
Source: Front Hum Neurosci. 2025 Sep 17;19:1618758. doi: 10.3389/fnhum.2025.1618758 (PMC12484017; doi:10.3389/fnhum.2025.1618758)

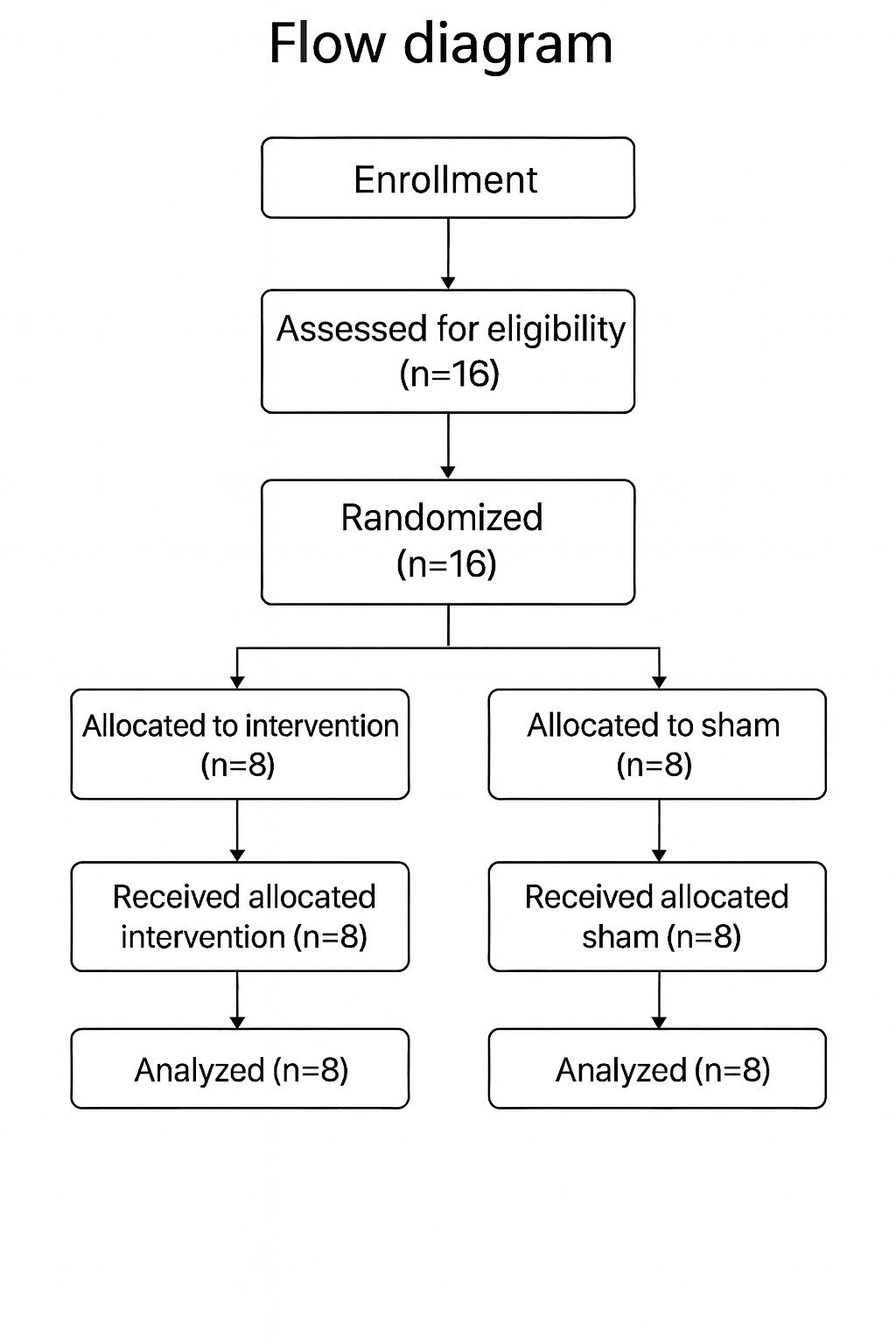

Supplement: Supplementary file 2 [file Image_1.jpeg]
